# Supplementary material for: Role of Pre-Farrow Natural Planned Exposure of Gilts in Shaping the Passive Antibody Response to Rotavirus A in Piglets
Source: Vaccines (Basel). 2023 Dec 18;11(12):1866. doi: 10.3390/vaccines11121866 (PMC10748143; doi:10.3390/vaccines11121866)
Supplement: Supplementary file 1 [file vaccines-11-01866-s001.zip › vaccines-2672226-supplementary.pdf]

Table S1: Longitudinal RVA detection levels in piglets' feces (5 piglets/litter) at multiple time points

Study groups	Sow Id.	RVA Ct values detected in piglet feces at different time points						
		Day 0	Day 7	Day 14	Day 21	Day 28	Day 35	Day 42
**Group 1**	40399					13.04	34.81	15.24
	38868					15.43	29.13	26.74
	41031					12.19	26.67	22.36
	41071					13.30	24.55	24.90
	41049		16.09	20.96	27.13	12.80	26.96	25.13
	41267					13.65	27.78	22.65
	40973					14.18	24.94	25.30
	40652					13.49	24.23	22.76
	40956					12.89	28.50	19.85
	41262					12.09	31.52	26.47
	41144					14.45	26.20	22.38
	41181					12.46	24.16	17.09
**Group 2**	41289					13.55	28.64	23.47
	41030					13.08	27.36	15.29
	41052					14.64	21.15	24.51
	40960					11.87	28.23	17.46
	41011					15.22	22.52	22.07
	40961					13.97	27.27	18.51
	41010					12.85	24.88	21.92
	40984					12.87	25.43	25.93
	41069					13.12	23.03	19.57
	41046					13.05	24.01	18.87
	40954					13.68	23.72	17.61
	41045					11.64	29.08	20.20
**Group 3**	41009					12.52	29.28	16.36
	41027					12.91	27.58	16.18
	40975					11.61	25.14	18.60
	40995					11.40	29.42	18.82
	41004					12.76	30.99	21.15
	40994					14.66	27.66	25.29
	41062					13.25	27.23	14.29
	41249					14.30	28.59	22.57
	41285					12.45	24.04	16.47
	40996		28.09	20.09	15.50	14.00	22.62	18.07
	41219					14.28	31.01	16.63
**Group 4**	40969					12.46	35.25	27.84

	41112	14.52	27.77	24.55
	41025	14.70	23.80	16.80
	41014	14.02	21.91	17.15
	41053	13.39	18.58	24.17
	41070	14.12	27.94	19.68
	41174	14.03	28.47	20.10
	40964	13.89	29.02	20.49
	40979	16.59	28.95	18.47
	41026		27.74	16.37
	41057	14.96	28.50	18.19
